# Cardio-Renal Metabolic Syndrome: An Integrated Approach to Prevention and Management

**DOI:** 10.7759/cureus.94134

**Published:** 2025-10-08

**Authors:** Adeshpal Singh, Hemanth Kesani, Sidhant Verma, Tareq Mohammed Saleh, Manju Rai

**Affiliations:** 1 Internal Medicine, Government Medical College, Amritsar, IND; 2 Internal Medicine, Narayana Medical College, Nellore, IND; 3 Internal Medicine, Suzhou Medical College, Suzhou, CHN; 4 Internal Medicine, Emilio Aguinaldo College School of Medicine, Manila, PHL; 5 Biotechnology, Shri Venkateshwara University, Gajraula, IND

**Keywords:** biomarkers, cardio-renal metabolic syndrome, cardiovascular disease, chronic kidney disease, glp-1 receptor agonists, insulin resistance, integrated care, metabolic syndrome, precision medicine, sglt2 inhibitors

## Abstract

Cardio-renal metabolic syndrome (CRMS), also termed cardiovascular-kidney-metabolic (CKM) syndrome, represents the convergence of metabolic risk factors, chronic kidney disease (CKD), and cardiovascular disease (CVD), underpinned by insulin resistance (IR), neurohormonal activation, oxidative stress, and chronic inflammation. This narrative review synthesizes current evidence on the epidemiology, pathophysiology, diagnostic approaches, prevention, and management of CRMS. Globally, CRMS prevalence continues to rise, driven by aging populations, urbanization, obesity, and diabetes, with disproportionate effects in low- and middle-income countries. The syndrome is associated with substantially increased morbidity and premature mortality, reflecting the synergistic effects of overlapping conditions. Advances in diagnostic evaluation, including novel biomarkers and imaging modalities, have improved early detection, but significant gaps remain. Preventive strategies emphasize lifestyle modification, dietary change, weight reduction, and smoking cessation, complemented by pharmacological therapies such as sodium-glucose cotransporter-2 (SGLT2) inhibitors, glucagon-like peptide-1 (GLP-1) receptor agonists, mineralocorticoid receptor antagonists (MRAs), and renin-angiotensin-aldosterone system (RAAS) blockade, all of which demonstrate cardio-renal protection. Non-pharmacological approaches, including bariatric surgery and renal replacement therapies (RRTs), add to the therapeutic armamentarium. Looking ahead, precision medicine, artificial intelligence (AI)-driven prognostic tools, and emerging biomarkers offer opportunities to refine risk stratification and treatment. An integrated, multidisciplinary framework is essential to reduce the escalating global burden and improve patient-centered outcomes in CRMS.

## Introduction and background

Cardio-renal metabolic syndrome (CRMS), also referred to as cardiovascular-kidney-metabolic (CKM) syndrome, represents a systemic disorder arising from the convergence of metabolic risk factors, chronic kidney disease (CKD), and cardiovascular disease (CVD) [1,2]. The concept evolved historically from two parallel lines of research: the recognition of metabolic syndrome as a clustering of obesity, diabetes, hypertension, and dyslipidemia [3], and the appreciation of bidirectional heart-kidney interactions in cardiorenal syndrome [4]. The modern CRMS construct integrates these domains into a unified framework that emphasizes inter-organ crosstalk and systemic pathobiology rather than isolated conditions [2,5].

The epidemiological burden of CRMS is considerable. Data from the Global Burden of Disease (GBD) study demonstrate that the prevalence of CVD nearly doubled from 271 million in 1990 to 523 million in 2019, with CVD-related deaths increasing to 18.6 million, driven largely by aging and rising cardiometabolic risk in low- and middle-income regions [6]. Parallel analyses show CKD to be among the fastest-growing contributors to years lived with disability worldwide, with substantial increases in prevalence and attributable mortality between 1990 and 2017 [7]. Metabolic syndrome, a core antecedent of CRMS, affects an estimated 20%-30% of adults globally, with higher prevalence in urbanized, high-income settings but rapidly rising rates across middle-income countries undergoing epidemiologic transition [8].

Regional and socioeconomic disparities strongly influence outcomes. Registry-based data and cohort studies consistently highlight worse CKD progression and cardiovascular outcomes among individuals with socioeconomic disadvantage, reflecting disparities in healthcare access, education, and environmental exposures [9]. Environmental studies further implicate air pollution and related contextual hazards as risk amplifiers for CKD and CVD, underscoring that CRMS is shaped not only by traditional risk factors but also by broader societal determinants [10].

More granular analyses emphasize the clinical implications of multimorbidity. In a nationwide Taiwanese cohort of over half a million individuals followed for 16.5 years, 71.5% met criteria for CRMS, with prevalence rising to nearly 90% in adults aged 55 years and older [5]. Each additional component of the syndrome, such as diabetes, hypertension, CKD, or dyslipidemia, was associated with progressively higher all-cause and cardiovascular mortality, shortening life expectancy by about three years per added component [5]. These findings underscore the synergistic nature of CRMS; the whole is more deleterious than the sum of its parts.

Together, these global and population-based data establish CRMS as a major driver of morbidity and mortality across diverse settings. The American Heart Association has emphasized early identification, integrated screening, and multidisciplinary care, spanning lifestyle modification, pharmacotherapy, and social determinants of health, as essential to mitigate the burden [2]. A fragmented approach to individual diseases is insufficient; only an integrated model targeting shared mechanisms such as insulin resistance (IR), inflammation, and neurohormonal activation can effectively address the escalating burden of CRMS worldwide.

The aim of this narrative review is to provide a comprehensive synthesis of current knowledge on CRMS, emphasizing its complex interplay between cardiovascular, renal, and metabolic dysfunction. Specifically, the review seeks to outline the epidemiology, shared risk factors, and underlying mechanisms that contribute to the development and progression of CRMS. It further aims to critically evaluate existing diagnostic strategies and risk stratification tools while examining preventive and therapeutic interventions that span lifestyle modification, pharmacological therapies, and interventional approaches. In addition, this review summarizes key evidence from major clinical trials and cohort studies, thereby offering insights into the effectiveness of various treatment modalities.

## Review

Methodology

This narrative review was conducted to comprehensively synthesize current evidence on CRMS, focusing on its pathophysiology, diagnostic strategies, preventive measures, and therapeutic interventions. A literature search was performed in PubMed/Medical Literature Analysis and Retrieval System Online (MEDLINE), Scopus, Web of Science, and Cochrane Library databases for studies published between January 2000 and August 2025. The search strategy combined free-text and MeSH terms, including “cardio-renal metabolic syndrome,” “cardiovascular-kidney-metabolic syndrome,” “chronic kidney disease,” “metabolic syndrome,” “insulin resistance,” “heart failure,” “SGLT2 inhibitors,” “GLP-1 receptor agonists,” “RAAS blockade,” “biomarkers,” and “integrated management.” Relevant articles were also identified from the references of key reviews and major clinical trials.

Inclusion criteria comprised peer-reviewed English-language studies involving adult human populations that examined epidemiology, mechanisms, diagnosis, prevention, or treatment of CRMS or overlapping cardiovascular, renal, and metabolic disorders. Experimental, observational, and interventional studies with significant clinical relevance were included. Exclusion criteria encompassed non-English publications, case reports, editorials, conference abstracts, and studies limited to isolated organ systems without an integrative focus.

Eligible studies were screened by title, abstract, and full text, and findings were synthesized qualitatively under thematic domains such as pathophysiology, biomarkers, and management approaches. Emphasis was placed on high-quality evidence from randomized controlled trials, prospective cohorts, and authoritative guidelines. The synthesis aims to provide a comprehensive, clinically oriented understanding of CRMS within an integrated cardio-renal-metabolic framework.

Pathophysiology and interplay of systems in CRMS

CRMS represents a convergence of metabolic, neurohormonal, and inflammatory disturbances that simultaneously damage the heart and kidneys. Central drivers include IR, visceral obesity, dyslipidemia, and low-grade inflammation, which together promote endothelial dysfunction, maladaptive remodeling, and progressive organ injury.

Adiposity and IR activate the renin-angiotensin-aldosterone system (RAAS) and the sympathetic nervous system (SNS), amplifying sodium retention, hypertension, and oxidative stress [11-12]. Normally, insulin facilitates endothelial nitric oxide (NO)-mediated vasodilation, but IR diverts signaling toward vasoconstriction and pro-inflammatory cascades, notably endothelin-1, impairing renal and myocardial microvascular function [13]. Oxidative stress further limits NO bioavailability, creating a cycle of vascular and renal injury [14].

Adipose tissue acts as an endocrine organ by secreting RAAS components and inflammatory cytokines; weight reduction has been shown to suppress this activation, linking obesity directly to hemodynamic stress [11]. Microneurography studies confirm heightened renal sympathetic activity in obesity, driving vasoconstriction, sodium reabsorption, and glomerular hyperfiltration that transitions into progressive decline [12]. Importantly, RAAS and SNS activation reinforce one another, potentiating oxidative and inflammatory pathways that promote concentric left ventricular remodeling and nephron loss.

Clinical cohorts validate these mechanisms. Albuminuria, reflecting systemic endothelial dysfunction, predicts incident heart failure (HF) even at high-normal ranges, independent of estimated glomerular filtration rate (eGFR) and traditional risk factors [15]. Similarly, lower eGFR and higher albuminuria jointly predict cardiovascular and all-cause mortality [16]. Obesity itself is an independent risk factor for kidney failure, as demonstrated in large community cohorts linking body mass index (BMI) with end-stage renal disease (ESRD) [17]. Within CKD, oxidative stress biomarkers such as F2-isoprostanes have been associated with accelerated renal decline [14].

Dyslipidemia further aggravates CRMS. Triglyceride-rich lipoproteins infiltrate the endothelium, worsen oxidative stress, and impair NO signaling, while high-density lipoprotein (HDL) dysfunction in CKD and IR states accelerates both vascular and renal inflammation [16].

Thus, CRMS emerges not as a simple comorbidity cluster but as a network disease with bidirectional organ crosstalk. Kidney injury (albuminuria, declining eGFR) predicts HF and ischemic events, while HF (via venous congestion, neurohormonal activation, and renal hypoperfusion) accelerates CKD progression [15-17].

Despite these insights, key gaps remain. Human studies often isolate single pathways, limiting causal clarity. Phenotypic diversity, ranging from lean IR to sarcopenic obesity or diabetic vs. non-diabetic CKD, suggests multiple CRMS endotypes are underrepresented in trials. Moreover, integrative biomarkers capturing combined RAAS, SNS, oxidative, and endothelial activity require validation. Addressing these gaps may allow precision, pathway-targeted strategies to disrupt the self-reinforcing heart-kidney injury loops central to CRMS.

Risk factors and clinical spectrum

CRMS aggregates common, often coexisting risk factors whose combined presence markedly elevates the risk of progressive cardiovascular and renal disease. Hypertension is both a cause and consequence within this syndrome: sustained blood pressure elevation promotes glomerulosclerosis and accelerates eGFR decline, while impaired renal function amplifies volume retention and neurohormonal activation that sustain hypertension. Large cohort analyses demonstrate a graded relationship between higher systolic/diastolic pressures and incident CKD and adverse renal outcomes, underscoring blood pressure control as a primary preventive target in CRMS [18,19].

Diabetes mellitus, particularly type 2 diabetes, remains one of the strongest clinical drivers of CRMS. Prospective community cohorts show that impaired glycemia and overt diabetes substantially increase the risk of new-onset CKD and eGFR deterioration, and diabetic individuals carry disproportionately high cardiovascular morbidity and mortality compared with non-diabetic peers [20]. The overlap of diabetes with microalbuminuria identifies patients at especially high risk for subsequent HF and atherosclerotic events, linking metabolic dysregulation to systemic microvascular injury [21].

CKD itself functions as an amplifier of cardiovascular risk within CRMS. Population-level estimates reveal that CKD prevalence and attributable morbidity are large and rising globally, and that even modest reductions in eGFR or modest increases in albuminuria confer markedly higher risks of cardiovascular events and death [18]. Albuminuria, in particular, emerges from longitudinal studies as a potent predictor of incident HF and cardiovascular outcomes independent of eGFR and traditional risk factors [21].

Dyslipidemia contributes to the clinical spectrum by promoting atherogenesis and microvascular dysfunction; in CKD populations, lipid abnormalities are common and modify cardiovascular risk. Interventional and outcome data from large randomized and cohort studies in CKD (for example, lipid-lowering trials that included patients with renal impairment) demonstrate that addressing atherogenic dyslipidemia reduces major atherosclerotic events, supporting lipid control as an integral component of CRMS management [17].

Obesity and adiposity-related phenotypes are prevalent upstream drivers. Large retrospective and longitudinal cohorts show graded associations between higher BMI and risk of ESRD, independent of baseline blood pressure and diabetes, evidence that excess adiposity contributes directly to renal hemodynamic stress, inflammation, and progression to renal failure [22]. Adiposity also potentiates IR and pro-inflammatory adipokine profiles that injure both the myocardium and the kidney.

Genetic and environmental predispositions modulate individual susceptibility and clinical heterogeneity. Genome-wide meta-analyses have identified hundreds of loci associated with kidney function, indicating polygenic determinants of renal vulnerability that likely interact with metabolic exposures to shape phenotype and progression [10]. Important environmental contributors, such as long-term exposure to fine particulate air pollution (PM2.5), have been implicated in large national cohorts as independent risk factors for incident CKD and faster progression to ESRD, illustrating nontraditional drivers of CRMS at the population level [23].

Clinically, CRMS presents with a spectrum from asymptomatic metabolic clustering (e.g., central obesity, dyslipidemia, elevated fasting glucose, microalbuminuria) to symptomatic HF, ischemic events, or progressive renal insufficiency. Diagnostic challenges include overlapping symptomatology, variable timing of organ-specific manifestations, under-recognition of low-grade albuminuria, and the need to integrate cardiac, renal, and metabolic biomarkers for risk stratification. Existing cohort evidence supports routine screening for albuminuria and eGFR in patients with metabolic risk factors and prompts early multimodal intervention to interrupt the syndrome’s trajectory.

Diagnostic evaluation and biomarkers in CRMS

**Figure 1:**
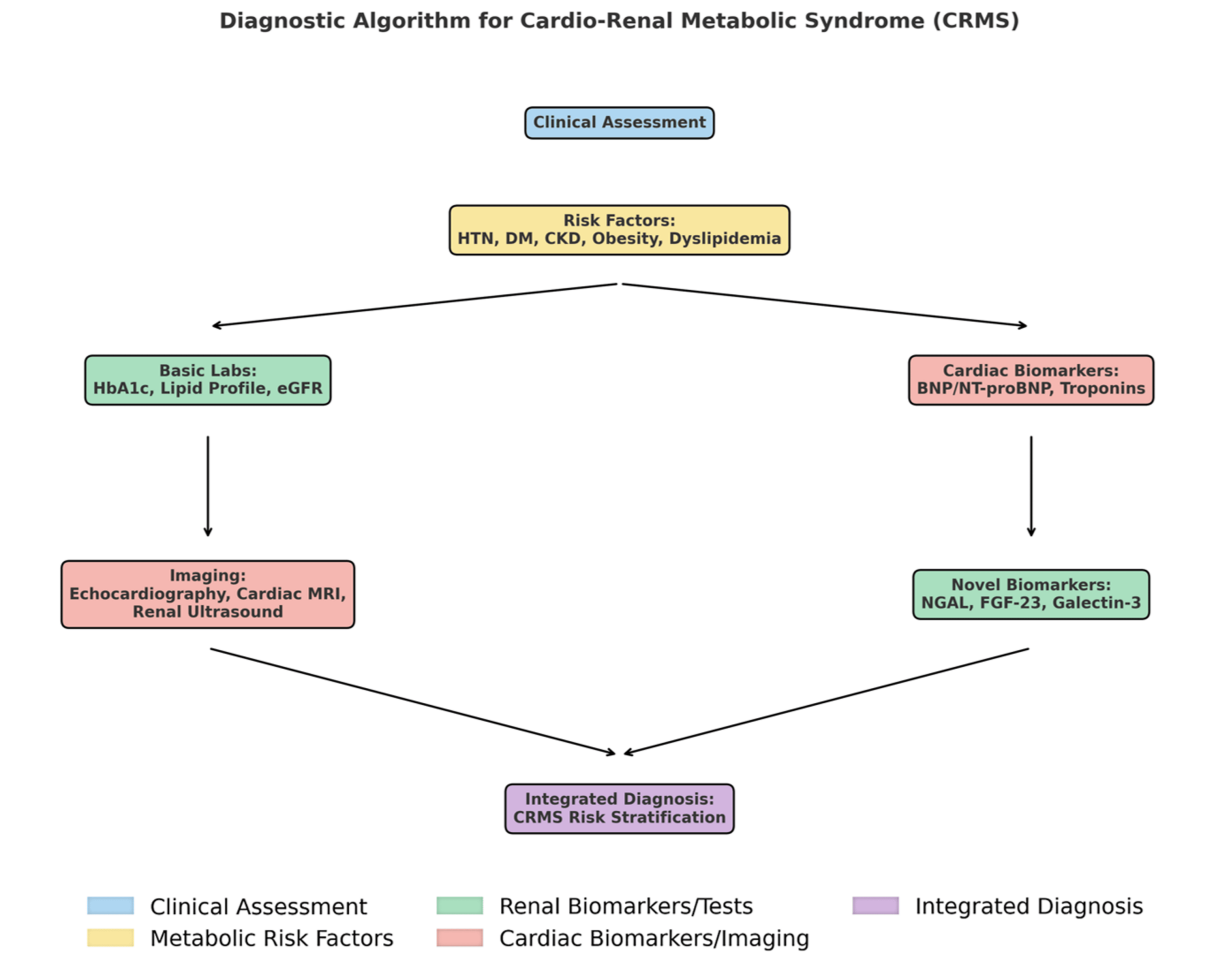
Cardio-renal metabolic syndrome (CRMS) diagnostic algorithm The schematic illustration of the diagnostic algorithm for CRMS, integrating clinical assessment, traditional laboratory tests, cardiac and renal biomarkers, and imaging modalities, alongside emerging biomarkers, to guide comprehensive risk stratification and diagnosis. HTN: hypertension; DM: diabetes mellitus; CKD: chronic kidney disease; eGFR: estimated glomerular filtration rate; NGAL: neutrophil gelatinase-associated lipocalin; FGF-23: fibroblast growth factor-23 Figure created by the author, Hemanth Kesani.

Diagnostic evaluation in CRMS necessitates a multimodal strategy that combines clinical assessment, imaging, and biomarkers to detect early dysfunction and stratify risk across the CRMS (Figure 1).

Traditional Biomarkers

Natriuretic peptides remain cornerstones of cardiovascular assessment. In the Chronic Renal Insufficiency Cohort (CRIC) study, Bansal et al. [24] evaluated 3,483 patients with CKD stages 2-4 and demonstrated that baseline NT-proBNP and high-sensitivity troponin T (hs-TnT) independently predicted incident HF, even after adjustment for traditional risk factors. Importantly, higher quartiles of these biomarkers were associated with a two- to three-fold increase in risk, underscoring their prognostic utility in CRMS.

Troponins provide complementary insights. Chesnaye et al. [25], in a Swedish cohort of 2,406 patients with advanced CKD followed over five years, showed that longitudinal increases in hs-TnT were strongly associated with all-cause mortality, highlighting the value of serial monitoring rather than single-timepoint testing.

Renal biomarkers are equally indispensable. eGFR and urinary albumin-to-creatinine ratio (UACR) are widely used, but recent evidence emphasizes their prognostic relevance in HF as well. Boorsma et al. [26], analyzing 2,312 HF patients from the EMPEROR-Reduced trial (short for Empagliflozin Outcome Trial in Patients with Chronic Heart Failure with Reduced Ejection Fraction), demonstrated that albuminuria correlated with systemic congestion and was independently predictive of adverse outcomes.

Emerging Biomarkers

Several novel biomarkers provide mechanistic insights and earlier detection. Bennett et al. [27] conducted a prospective study of 196 children undergoing cardiac surgery and found that elevated urine neutrophil gelatinase-associated lipocalin (NGAL) within two hours postoperatively predicted the severity and duration of acute kidney injury (AKI), with an area under the curve (AUC) of 0.95 for severe AKI. This early signal highlights its role in identifying tubular injury prior to creatinine rise.

Fibroblast growth factor-23 (FGF-23), a regulator of phosphate metabolism, has been implicated in cardiovascular remodeling. In the Multi-Ethnic Study of Atherosclerosis (MESA), Kestenbaum et al. [28] evaluated 6,547 community participants without prior CVD and reported that higher FGF-23 levels were associated with increased risks of left ventricular hypertrophy and incident HF over a median 7.6-year follow-up.

Galectin-3, a marker of fibrosis, has also been validated. Van der Velde et al. [29] examined 1,196 patients with chronic HF in the Controlled Rosuvastatin Multinational Trial in Heart Failure (CORONA) and Coordinating Study Evaluating Outcomes of Advising and Counseling in Heart Failure (COACH) cohorts and showed that rising galectin-3 levels over time significantly predicted hospitalizations and mortality, even after adjustment for renal function.

Imaging Modalities

Imaging augments biomarker data. Kalogeropoulos et al. [30] demonstrated in 5,613 older adults from the Cardiovascular Health Study that echocardiographic measures of diastolic dysfunction, when combined with natriuretic peptides, improved the prediction of incident HF. Similarly, Konst et al. [31], in a prospective cohort of 388 patients with myocardial infarction with non-obstructive coronary arteries (MINOCA), showed that cardiac magnetic resonance (CMR) with late gadolinium enhancement provided incremental prognostic information over biomarkers during 10 years of follow-up.

Despite these advances, several diagnostic gaps remain. Interpretation of biomarkers such as NT-proBNP and galectin-3 is confounded by reduced renal clearance, and standardized thresholds across CKD stages are lacking. Moreover, while emerging biomarkers (NGAL, FGF-23, and galectin-3) show strong associations with outcomes, few trials have evaluated whether biomarker-guided interventions improve prognosis in CRMS. Future work should focus on multimarker panels that integrate cardiac, renal, and metabolic biomarkers with imaging modalities, validated through large randomized or pragmatic trials.

Integrated preventive strategies for CRMS

The prevention of CRMS requires a multifaceted and integrated approach that addresses the shared risk factors of cardiovascular disease, CKD, and metabolic dysfunction. Lifestyle modification remains the cornerstone, reinforced by robust evidence from prospective cohort studies and randomized prevention trials.

Lifestyle and Behavioral Interventions

Large prospective cohorts have consistently demonstrated the protective effects of healthy lifestyle behaviors. In the Nurses’ Health Study, Hu et al. followed >84,000 women free of cardiovascular and renal disease at baseline and showed that adherence to five low-risk lifestyle factors (healthy diet, physical activity, normal body weight, no smoking, and moderate alcohol) was associated with a ~72% lower risk of developing type 2 diabetes and markedly reduced cardiovascular risk over ~20 years [32]. Similarly, analyses from the Malmö Diet and Cancer cohort linked adherence to a health-conscious lifestyle score with lower risk of CKD progression and cardiovascular mortality in a community sample followed longitudinally [33].

Smoking cessation is a critical preventive measure. A meta-analysis of prospective cohorts by Xia et al. synthesized data from large population studies and found that cigarette smoking accelerates kidney function decline and heightens cardiovascular morbidity; importantly, risk attenuated after smoking cessation over subsequent years [34]. These data underscore the need to integrate structured cessation programs into CRMS care pathways.

Dietary Modification

Dietary quality exerts a strong influence on both cardiovascular and renal outcomes. The Prevención con Dieta Mediterránea (PREDIMED) randomized trial enrolled 7,447 high-cardiovascular-risk adults and randomized them to a Mediterranean diet (supplemented with extra-virgin olive oil or nuts) or control; over a median of 4.8 years, the Mediterranean diet arms had 30% fewer major cardiovascular events, with secondary analyses suggesting slower eGFR decline versus controls [35]. The Dietary Approaches to Stop Hypertension (DASH) diet feeding trial randomized 459 adults with elevated blood pressure to a DASH or control diet and demonstrated rapid and clinically meaningful reductions in systolic and diastolic blood pressure within eight weeks, findings that have been linked to lower long-term risks of HF, stroke, and CKD in cohort follow-up studies [36-37].

Exercise and Weight Control

Structured exercise and weight loss interventions reduce cardiometabolic risk. The Diabetes Prevention Program randomized 3,234 adults with impaired glucose tolerance to intensive lifestyle modification (target: 7% weight loss, ≥150 min/week activity) versus metformin or usual care; lifestyle intervention cut progression to diabetes by 58% at 3 years, with durable benefits at 10 years, improvements that translate into reduced cardiorenal risk [38]. The Look AHEAD (Action for Health in Diabetes) trial randomized 5,145 overweight/obese adults with type 2 diabetes to intensive lifestyle intervention versus usual care and produced sustained weight loss and improvements in glycemia and other risk factors over years, though the intervention did not significantly reduce major cardiovascular events in the primary analysis; nonetheless, it demonstrated the feasibility and risk-factor benefits of comprehensive weight programs relevant to CRMS prevention [39].

Public Health and Multidisciplinary Approaches

Population-level strategies are essential to curb CRMS. The Prospective Urban Rural Epidemiology (PURE) study followed more than 135,000 participants across 18 countries and highlighted profound global disparities in diet quality, risk-factor prevalence, and access to care, providing evidence supporting policies to improve food environments and healthcare access [40]. Integrated primary care models using the Chronic Care Model combine lifestyle coaching, multidisciplinary teams, and systematic risk monitoring and have shown improved blood-pressure control, glycemic indices, and other intermediate outcomes relevant to CRMS prevention [41].

Randomized trials and long-term cohorts consistently support lifestyle interventions (diet, exercise, and weight control), smoking cessation, and population-level strategies as foundational to CRMS prevention. Early, multidisciplinary programs that combine behavioral interventions with structural, policy-level changes are required to reduce the global and inequitable burden of cardio-renal metabolic disease.

Therapeutic approaches to CRMS

The management of CRMS requires a comprehensive strategy that integrates both pharmacological and non-pharmacological approaches. Given the shared pathophysiology of metabolic dysregulation, vascular injury, and renal impairment, therapeutic interventions should target multiple organ systems simultaneously. Evidence from large-scale randomized trials and observational cohorts provides the basis for optimal treatment selection.

Pharmacological Therapies

RAAS inhibitors: RAAS blockade remains a cornerstone of therapy in CRMS due to its dual cardiovascular and renal benefits. In the Heart Outcomes Prevention Evaluation (HOPE) trial, Yusuf et al. randomized 9,297 patients at high cardiovascular risk to ramipril or placebo and demonstrated a 22% reduction in myocardial infarction, stroke, or cardiovascular death with ramipril [42]. Similarly, the RENAAL trial (short for Reduction of Endpoints in NIDDM with the Angiotensin II Antagonist Losartan) in 1,513 patients with type 2 diabetes and nephropathy showed that losartan significantly reduced the risk of doubling serum creatinine and progression to ESRD compared with placebo [43]. These findings underscore the renoprotective and cardioprotective effects of RAAS inhibition.

Sodium-glucose cotransporter-2 (SGLT2) inhibitors: SGLT2 inhibitors have transformed management of CRMS by simultaneously improving glycemia, reducing cardiovascular events, and preserving renal function. The Empagliflozin Cardiovascular Outcome Event Trial in Type 2 Diabetes Mellitus Patients (EMPA-REG)-OUTCOME trial enrolled 7,020 patients with type 2 diabetes and established cardiovascular disease, finding that empagliflozin reduced cardiovascular death by 38% and hospitalization for HF by 35% compared with placebo [44]. In the Dapagliflozin And Prevention of Adverse Outcomes in Chronic Kidney Disease (DAPA-CKD) trial, Heerspink et al. studied 4,304 patients with CKD (with and without diabetes) and found that dapagliflozin reduced the composite of sustained eGFR decline, ESRD, or renal/cardiovascular death by 39% [45]. These benefits extended across diverse patient populations, making SGLT2 inhibitors essential in CRMS management.

Glucagon-like peptide-1 (GLP-1)receptor agonists: GLP-1 receptor agonists provide complementary cardiometabolic benefits, particularly in patients with type 2 diabetes and obesity. The Liraglutide Effect and Action in Diabetes: Evaluation of cardiovascular outcome Results (LEADER) trial randomized 9,340 high-risk diabetic patients to liraglutide or placebo and observed a 13% reduction in major adverse cardiovascular events (MACE) and a significant reduction in nephropathy progression [46]. Similarly, the Semaglutide Unabated Sustainability in Treatment of Type 2 Diabetes Outcome Study 6 (SUSTAIN-6) trial with semaglutide in 3,297 patients demonstrated a 26% reduction in MACE [47]. These agents are particularly valuable in obese or IR CRMS patients.

Statins: Lipid-lowering with statins has long been proven effective in reducing cardiovascular risk in metabolic syndrome and CKD. The Study of Heart and Renal Protection (SHARP) trial randomized 9,270 patients with CKD to simvastatin plus ezetimibe or placebo, showing a 17% reduction in major atherosclerotic events without a significant effect on progression to dialysis [23]. Statins remain first-line agents in CRMS patients with dyslipidemia, though their renoprotective effects are modest.

Mineralocorticoid receptor antagonists (MRAs): Finerenone, a novel nonsteroidal MRA, has shown promise in reducing both renal and cardiovascular events in patients with diabetic kidney disease. The Finerenone in Reducing Kidney Failure and Disease Progression in Diabetic Kidney Disease (FIDELIO-DKD) trial randomized 5,734 patients with diabetic CKD and found finerenone reduced the composite renal outcome by 18% and cardiovascular events by 14% compared with placebo [48]. These benefits were confirmed in the Finerenone in Reducing Cardiovascular Mortality and Morbidity in Diabetic Kidney Disease (FIGARO-DKD) trial, highlighting the role of MRAs as adjunct therapy in CRMS.

Non-pharmacological Approaches

Bariatric surgery: For obese patients with refractory metabolic syndrome, bariatric (metabolic) surgery offers substantial and durable improvements in cardiometabolic health and meaningful renal benefits when carefully selected and managed. Large prospective cohorts and population studies demonstrate long-term reductions in all-cause and cardiovascular mortality after gastric bypass and other bariatric procedures. Adams and colleagues reported significantly lower long-term mortality, including fewer deaths from diabetes and cardiovascular disease, in patients undergoing gastric bypass versus matched severely obese controls [49]. Beyond survival, contemporary observational and registry analyses consistently show that bariatric surgery is associated with slower decline in kidney function and a lower risk of major renal endpoints (≥30% eGFR decline, doubling of serum creatinine, or progression to ESRD) compared with non-operative controls; a detailed review and pooled analyses summarize hazard reductions approaching ~40%-60% for clinically important renal outcomes [50].

Mechanistically, weight loss and metabolic improvements after surgery reduce glomerular hyperfiltration, lower intrarenal inflammation and oxidative stress, ameliorate albuminuria, and improve insulin sensitivity and blood pressure control, all processes central to the CRMS. Short- and medium-term cohort studies report increases in measured and eGFR and reductions in albuminuria in many patients with baseline CKD, although the magnitude of benefit varies by baseline kidney function, procedure type, and degree of weight loss [51]. Importantly, perioperative and longer-term risks (AKI, nephrolithiasis, nutritional deficiencies, and procedure-specific complications) must be anticipated and mitigated with preoperative risk stratification, perioperative nephrology input for patients with CKD, and structured long-term follow-up. Recent studies also highlight better transplant access and lower mortality among selected patients with obesity and advanced kidney disease who undergo metabolic surgery [52].

Renal replacement therapies (RRTs): For advanced CKD in CRMS, RRTs such as dialysis and transplantation remain critical. Observational registry data (United States Renal Data System (USRDS) Annual Data Report) consistently demonstrate that kidney transplantation, compared with dialysis, confers a 50%-70% lower risk of mortality and substantial improvements in cardiovascular outcomes in patients with cardiorenal metabolic comorbidity [53].

Additional recent studies further quantify and refine this benefit, especially in terms of MACEs, long-term survival, and modality effects among dialysis populations. A large matched-cohort study from South Korea of 4,156 kidney transplant recipients (KTRs) vs. dialysis patients, followed for ~4.7 years, found de novo MACEs occurred at rates of 3.7 versus 21.7 per 1,000 person-years, respectively. After adjustment, transplant recipients had an 84% lower risk of MACE compared to those remaining on dialysis. All-cause mortality was also much lower in the transplant group [54].

Other registry/data-linkage work from the Australia-New Zealand Dialysis and Transplant (ANZDATA) cohort used multi-state modeling to evaluate cardiovascular mortality in patients initiated on kidney replacement therapy. It showed that cardiovascular death probabilities among transplant recipients were far lower than among dialysis patients at five, 10, and 15 years after therapy initiation-e.g., cardiovascular mortality at five years post-KRT was 0.4% in transplant recipients vs. 2.7% in all KRT patients, including dialysis; by 15 years, the gap widened substantially [55].

Furthermore, while dialysis modality matters, a 2025 retrospective cohort (NephroCare Europe, Middle East, and Africa (EMEA)) comparing high-volume hemodiafiltration vs conventional hemodialysis in 85,000 patients found a significant reduction in both all-cause and cardiovascular mortality with hemodiafiltration [56]. This underscores that even in non-transplant RRT, the choice of modality can modulate cardiovascular risk.

Multidisciplinary Care and Comparative Effectiveness

Given the multisystem nature of CRMS, multidisciplinary care involving cardiology, nephrology, endocrinology, bariatric surgery, nutrition/dietetics, and vascular medicine is essential. Integrated care models that combine lifestyle interventions, surgical approaches, and multiple pharmacological agents are increasingly shown to produce additive or even synergistic benefits in reducing both cardiovascular and renal morbidity and mortality.

Recent comparative effectiveness research reinforces that combining agents that target different mechanistic axes yields superior outcomes to monotherapy. A 2024 meta-analysis by Neuen et al. showed that GLP-1 receptor agonists, whether used alone or in combination with SGLT2 inhibitors, reduce the risk of MACE by ~20-25%, HF hospitalizations, and composite kidney outcomes (≥50% eGFR decline, kidney failure, or death from kidney causes), relative to placebo. Notably, the magnitude of benefit was consistent whether or not an SGLT2 inhibitor was already in use [57]. 

Another recent study summarized the effects of SGLT2 inhibitors across diverse populations (diabetes, HF, CKD, non-diabetic kidney disease), showing that they confer substantial reductions in cardiovascular death, HF hospitalizations, and kidney composite outcomes even in non-diabetic CKD. This supports broader use across the spectrum of CRMD [58].

Major clinical trials and cohort studies evaluating therapeutic approaches in CRMs are summarized in Table 1.

**Table 1:** Major clinical trials and cohort studies evaluating therapeutic approaches in CRMS SHARP: Study of Heart and Renal Protection; CKD: chronic kidney disease; T2DM: type 2 diabetes mellitus; HOPE: Heart Outcomes Prevention Evaluation; MI: myocardial infarction; CV: cardiovascular; RENAAL: Reduction of Endpoints in NIDDM with the Angiotensin II Antagonist Losartan; ESRD: end-stage renal disease; EMPA-REG: Empagliflozin Cardiovascular Outcome Event Trial in Type 2 Diabetes Mellitus Patients; HF: heart failure; DAPA-CKD: Dapagliflozin And Prevention of Adverse Outcomes in Chronic Kidney Disease; eGFR: estimated glomerular filtration rate; LEADER: Liraglutide Effect and Action in Diabetes: Evaluation of cardiovascular outcome Results; MACE: major adverse cardiovascular events; SUSTAIN-6: Semaglutide Unabated Sustainability in Treatment of Type 2 Diabetes Outcome Study 6; FIDELIO-DKD: Finerenone in Reducing Kidney Failure and Disease Progression in Diabetic Kidney Disease; USRDS: United States Renal Data System; CRMS: cardio-renal metabolic syndrome

Trial/Study	Patients	Intervention	Primary Outcomes	Key Results
SHARP (Baigent et al., 2011) [23]	9,270 patients with CKD	Simvastatin + ezetimibe vs placebo	Major atherosclerotic events	17% ↓ in vascular events; no significant effect on dialysis progression
Diabetes Prevention Program (Knowler et al., 2002) [38]	3,234 overweight adults with impaired glucose tolerance	Lifestyle intervention vs. metformin vs. placebo	Progression to T2DM	Lifestyle ↓ diabetes incidence by 58%, metformin by 31%
HOPE (Yusuf et al., 2000) [42]	9,297 high-risk cardiovascular patients (diabetes, vascular disease, or hypertension)	Ramipril vs. placebo	Composite of MI, stroke, CV death	22% reduction in primary outcome with ramipril
RENAAL (Brenner et al., 2001) [43]	1,513 patients with T2DM and nephropathy	Losartan vs. placebo	Doubling of serum creatinine, ESRD, and death	Losartan reduced renal endpoints by 16%, lowered proteinuria
EMPA-REG OUTCOME (Zinman et al., 2015) [44]	7,020 patients with T2DM and established CVD	Empagliflozin vs. placebo	CV death, MI, stroke	38% ↓ CV death, 35% ↓ HF hospitalization
DAPA-CKD (Heerspink et al., 2020) [45]	4,304 patients with CKD (with/without diabetes)	Dapagliflozin vs. placebo	Sustained eGFR decline, ESRD, renal/CV death	39% risk reduction in renal/CV composite outcome
LEADER (Marso et al., 2016) [46]	9,340 patients with T2DM at high CV risk	Liraglutide vs.placebo	MACE	13% ↓ MACE, ↓ nephropathy progression
SUSTAIN-6 (Marso et al., 2016) [47]	3,297 patients with T2DM at CV risk	Semaglutide vs. placebo	MACE	26% ↓ MACE, favorable renal outcomes
FIDELIO-DKD (Bakris et al., 2020) [48]	5,734 patients with T2DM and CKD	Finerenone vs. placebo	Renal composite (ESRD, renal death, eGFR decline) + CV events	18% ↓ renal outcomes, 14% ↓ CV events
USRDS registry data (2020) [53]	CKD/ESRD patients in the U.S. registry	Dialysis vs kidney transplantation	Mortality, CV outcomes	Transplantation ↓ mortality by 50–70% vs dialysis

Future directions and emerging therapies in CRMS

Advances in CRMS management are increasingly shifting toward individualized and technology-driven strategies. Precision medicine, artificial intelligence (AI), novel biomarkers, and next-generation therapeutics represent promising frontiers, though significant gaps remain in their clinical application.

Precision Medicine Approaches

Patient heterogeneity in CRMS poses a major therapeutic challenge. Efforts to stratify patients based on genomic, proteomic, and metabolomic profiles have shown promise in guiding therapy. In the CARDIoGRAMplusC4D consortium study (short for Coronary ARtery DIsease Genome-wide Replication and Meta-analysis plus The Coronary Artery Disease (C4D) Genetics), Nikpay et al. analyzed genome-wide data from over 60,000 patients with coronary artery disease and highlighted shared loci associated with both cardiovascular and metabolic traits [59]. Similarly, proteomic profiling in cohorts with CKD has identified circulating proteins predictive of progression and treatment response, paving the way for targeted therapy [60].

AI-Driven Prognostic Models

AI and machine learning are increasingly applied to large clinical and biomarker datasets to improve risk stratification in CRMS. Oo et al. developed a deep learning-based survival model using electronic health records of more than 100,000 patients, demonstrating superior prediction of cardiovascular mortality compared with conventional scores [61]. In nephrology, Tangri et al. validated the Kidney Failure Risk Equation in diverse populations, showing its capacity to predict ESRD progression with high accuracy [62]. Such models may enable earlier intervention and more efficient resource allocation.

Novel Biomarkers

Beyond traditional risk factors, new biomarkers are being evaluated in early-phase studies. Soluble ST2 and growth differentiation factor-15 (GDF-15) have shown incremental prognostic value in patients with both HF and CKD [63]. Circulating microRNAs, including miR-21 and miR-126, have been linked to vascular injury and renal fibrosis in prospective biomarker cohorts [64]. These tools may enhance the diagnosis and monitoring of disease activity, though clinical validation remains limited.

Next-Generation Therapeutics

Emerging pharmacological approaches include agents targeting inflammation and fibrosis. The Canakinumab Anti-inflammatory Thrombosis Outcomes Study (CANTOS) trial demonstrated that canakinumab, an IL-1β inhibitor, reduced recurrent cardiovascular events in patients with prior myocardial infarction, though renal outcomes were not directly assessed [65]. Novel antifibrotic therapies, including pentraxin-2 analogs and NLRP3 inflammasome inhibitors, are under investigation in early-phase trials for diabetic kidney disease [66].

Future management of CRMS will likely be defined by integration of precision medicine, advanced prognostic modeling, and novel therapeutics. However, significant gaps exist in translating these approaches into routine practice, particularly in validating biomarkers, ensuring equitable access to AI-driven tools, and assessing the long-term safety of emerging agents. Bridging these gaps through collaborative, multidisciplinary research is essential to realize the full potential of these innovations.

## Conclusions

CRMS epitomizes the convergence of cardiovascular, renal, and metabolic dysfunction, driven by IR, neurohormonal activation, oxidative stress, and inflammation. The coexistence of diabetes, obesity, hypertension, dyslipidemia, and CKD significantly amplifies morbidity and mortality, with prevalence rising worldwide and particularly affecting vulnerable populations. Despite advances in biomarkers and imaging, early diagnosis and risk prediction remain challenging.

Management of CRMS requires an integrated, multidisciplinary approach. Lifestyle interventions, weight control, dietary modification, and smoking cessation remain central to prevention, while pharmacotherapies such as SGLT2 inhibitors, GLP-1 receptor agonists, mineralocorticoid receptor antagonists, and RAAS blockade have demonstrated robust cardiovascular and renal benefits. However, the heterogeneous presentations of CRMS demand personalized strategies that transcend siloed disease management. Future directions, including precision medicine, AI-driven risk stratification, and novel therapeutics, hold promise for reducing its growing global burden and improving patient-centered outcomes.
